# The Histone Deacetylase Inhibitor Valproic Acid Exerts a Synergistic Cytotoxicity with the DNA-Damaging Drug Ellipticine in Neuroblastoma Cells

**DOI:** 10.3390/ijms19010164

**Published:** 2018-01-05

**Authors:** Tereza Cerna, Jan Hrabeta, Tomas Eckschlager, Eva Frei, Heinz H. Schmeiser, Volker M. Arlt, Marie Stiborová

**Affiliations:** 1 Department of Biochemistry, Faculty of Science, Charles University, Albertov 2030, CZ-128 43 Prague 2, Czech Republic; cernat10@natur.cuni.cz (T.C.); evafrei@t-online.de (E.F.); 2 Department of Pediatric Hematology and Oncology, 2nd Faculty of Medicine, Charles University and University Hospital Motol, V Uvalu 84/1, CZ-150 06 Prague 5, Czech Republic; janhrabeta@gmail.com (J.H.); tomas.eckschlager@lfmtol.cuni.cz (T.E.); 3 Division of Radiopharmaceutical Chemistry, German Cancer Research Center (DKFZ), Im Neuenheimer Feld 280, 69120 Heidelberg, Germany; h.schmeiser@dkfz-heidelberg.de; 4 Analytical and Environmental Sciences Division, MRC-PHE Centre for Environment and Health, King’s College London, London WC2R 2LS, UK; volker.arlt@kcl.ac.uk; 5 NIHR Health Protection Research Unit in Health Impact of Environmental Hazards at King’s College London in Partnership with Public Health England, London WC2R 2LS, UK

**Keywords:** neuroblastoma, ellipticine, valproate, DNA damage, acetylation of histones, apoptosis

## Abstract

Neuroblastoma (NBL) originates from undifferentiated cells of the sympathetic nervous system. Chemotherapy is judged to be suitable for successful treatment of this disease. Here, the influence of histone deacetylase (HDAC) inhibitor valproate (VPA) combined with DNA-damaging chemotherapeutic, ellipticine, on UKF-NB-4 and SH-SY5Y neuroblastoma cells was investigated. Treatment of these cells with ellipticine in combination with VPA led to the synergism of their anticancer efficacy. The effect is more pronounced in the UKF-NB-4 cell line, the line with *N-myc* amplification, than in SH-SY5Y cells. This was associated with caspase-3-dependent induction of apoptosis in UKF-NB-4 cells. The increase in cytotoxicity of ellipticine in UKF-NB-4 by VPA is dictated by the sequence of drug administration; the increased cytotoxicity was seen only after either simultaneous exposure to these drugs or after pretreatment of cells with ellipticine before their treatment with VPA. The synergism of treatment of cells with VPA and ellipticine seems to be connected with increased acetylation of histones H3 and H4. Further, co-treatment of cells with ellipticine and VPA increased the formation of ellipticine-derived DNA adducts, which indicates an easier accessibility of ellipticine to DNA in cells by its co-treatment with VPA and also resulted in higher ellipticine cytotoxicity. The results are promising for in vivo studies and perhaps later for clinical studies of combined treatment of children suffering from high-risk NBL.

## 1. Introduction

Neuroblastoma (NBL) represents the commonest extracranial solid tumor of children. In child organisms, NBL cells can spontaneously regress or differentiate; they can naturally deteriorate, develop to benign ganglioneuroma, or grow continuously becoming speedily lethal depending on the NBL biological type [1,2]. These processes might influence the efficacy of therapy, especially the replay on apoptosis developed during chemotherapy. Despite advances in cancer diagnosis and therapy, patients suffering from high-risk NBL possess long-term survival rates of less than 40% [3]. Therefore, novel drugs and treatment procedures are necessary to improve the efficiency of treatment of the disease.

An important function for epigenetic mechanisms in evolution of NBL was found in several studies [4,5,6]. It has been demonstrated that carcinogenic processes in NBL need not result solely from genetic changes; however they can also be developed by numerous epigenetic mechanisms such as hyperacetylation and re-expression of growth regulatory genes [4]. DNA hypermethylation and gene silencing are frequently connected with the abundancy of histones modified by their deacetylation in NBL [6,7,8]. In total, there are about 75 genes described as epigenetically affected in several NBL cell lines and/or NBL samples [6]. Histone modifications include the lysine acetylation status of the core histones H3 and H4, which influence chromatin condensation. Such changes affect transcription of several genes together with upregulation of numerous antioncogenes and genes participating in repair of DNA [9]. Hence, epigenetic mechanisms have arisen to be new therapeutic goals largely investigated in multiple studies including NBL. Acetylation of histones is maintained by the balance between activities of two types of enzymes, namely histone acetyltransferases (HATs) and histone deacetylases (HDACs) [6]. HDACs also function in posttranscriptional alterations of numerous regulatory non-histone proteins, for example several transcription factors, chaperones or signaling factors [10].

A variety of substances that inhibit HDACs have been shown to be antitumor agents [11,12]. In the case of one of the HDAC inhibitors, valproate (VPA), it was demonstrated that that can decrease the aggressiveness in bladder cancer however not in prostate cancer cells [13]. Further, stimulation of SH-SY5Y and SK-N-BE NBL cells with VPA led to increased cell death and phenotypic changes associated with cell differentiation, that is, neurite extension and up-regulation of neuronal markers [14]. VPA is currently used for long-term treatment of epilepsy in both adults and children. The drug also possesses antitumoral activity, which has led to several preclinical studies showing that VPA induces loss of proliferative capacity and promotes differentiation of several tumor cell types [12]. The exact mechanism of the anticancer effect of VPA is however still unclear. VPA not only suppresses tumor growth and metastatic processes, but it also induces tumor differentiation and apoptosis. Several mechanisms might be relevant for the biological activity of VPA: (i) it increases the DNA binding of activating protein-1 (AP-1) transcription factor and the expression of genes regulated by the extracellular-regulated kinase (ERK)-AP-1 pathway, (ii) VPA down-regulates protein kinase C (PKC) activity, (iii) inhibits glycogen synthase kinase-3beta (GSK-3beta), (iv) acts as a negative regulator of the Wnt signaling pathway, and (v) activates the peroxisome proliferator-activated receptors (PPAR) gamma and delta [12,15].

A number of clinical trials studying the effectiveness of individual inhibitors of HDACs or that of these compounds in combined chemotherapy with other antitumor drugs and/or radiotherapy showed that they can produce additive or synergistic effects when utilized together with several cytostatics or ionizing radiation [12,15,16,17,18,19,20,21,22,23,24,25,26,27,28]. Recently, we have found that exposure of the UKF-NB-4 cell line derived from high-risk NBL tumor to the DNA-damaging drugs cisplatin or etoposide in a combination with an inhibitor of class I and IIA HDACs, VPA [29], resulted in a synergistic cytotoxic effect [25,26].

In those studies, potential mechanisms responsible for the increased cytotoxicity of cisplatin and etoposide were investigated and the results suggested mechanisms dependent on initiation of caspase-3-dependent apoptosis. The synergism was only produced when VPA was used together with antitumor drugs targeted at cellular DNA; this HDAC inhibitor is not capable of potentiating the cytotoxic effect of the antitumor agent vincristine, which is the drug acting as a mitotic inhibitor and does not damage DNA directly. Interestingly, the increase in cytotoxicity of cisplatin and etoposide in UKF-NB-4 caused by VPA was dictated by the sequence of administration of these drugs and VPA; the increased cytoxicity resulted only from either simultaneous exposure to these drugs with VPA or after the pretreatment of the cells with cisplatin and etoposide before their exposure to VPA. The synergism of VPA with cisplatin or etoposide correlated with the degree of acetylation of histones H3 and H4. However, exact mechanisms of these processes require further investigations.

Here we further investigated the potentiating influence of VPA on cytotoxic effects caused by DNA-damaging drugs in NBL cells. Because the DNA-damaging drug ellipticine efficiently inhibits the growth of NBL cells and produces the initiation of apoptosis in the cells [23,30,31,32,33,34], we investigated the influence of VPA on ellipticine-induced cytotoxicity. The DNA-damaging mechanisms leading to ellipticine cytotoxicity are produced by its ability to intercalate into DNA [35,36,37], to act as the inhibitor of topoisomerase II generating DNA double-strand breaks [38], and predominantly by its capability of covalent binding to DNA (formation of covalent DNA adducts) when activated by cytochrome P450 (CYP) enzymes and/or peroxidases [30,31,39,40,41,42,43,44,45]). Two types of NBL cells differing in *N-myc* gene status were used in our experiments, because this genotype can influence the pathogenesis of NBL [46]; the UKF-NB-4 cells with and the SH-SY5Y cells without *N-myc* amplification were utilized. Both tested cell lines were found to differ significantly in sensitivity to VPA and several cytostatics; the SH-SY5Y cell line is less sensitive than UKF-NB-4 cells [47]. Therefore, we examined whether cell sensitivity to ellipticine, which is promising for treatment of high-risk NBL, might be improved by co-treatment of these SH-SY5Y cells with ellipticine and VPA. We evaluated the combined effects of VPA with ellipticine on NBL cells under various treatment conditions by studying the pro-apoptotic efficacy of these chemotherapeutics. We investigated the mechanisms resulting from apoptosis emphasizing the anticancer effects of VPA, and assessed the influence of VPA on ellipticine-induced DNA damage by measuring the production of double-strand-breaks and formation of covalent DNA adducts. Our results suggest that integrating VPA into therapy of high-risk NBL can increase treatment efficiency.

## 2. Results

### 2.1. VPA Enhances Cytotoxicity of Ellipticine in Human UKF-NB-4 and SH-SY5Y NBL Cells

**Figure 1:**
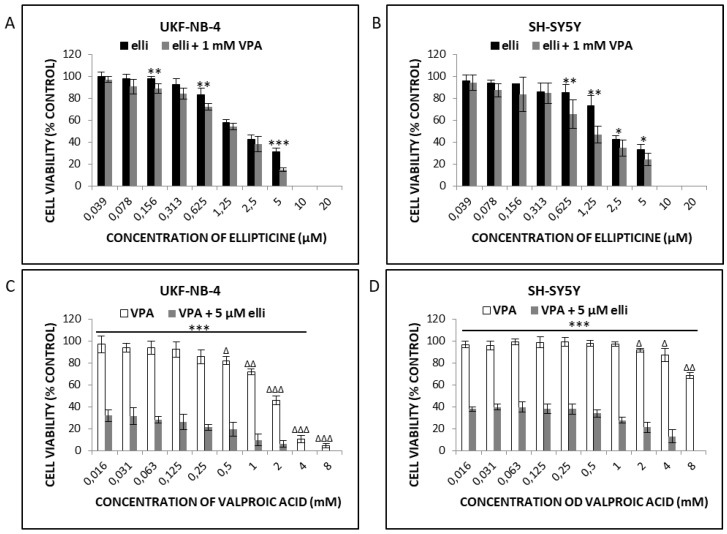
Cytotoxicity (viable cells % control) of ellipticine (elli) and/or valproic acid (VPA) or their combination in UKF-NB-4 (**A**,**C**) and SH-SY5Y (**B**,**D**) cells after 48 h exposure to drugs, measured by the MTT assay. Values are mean ± SD from three independent experiments. Panels (**A**–**D**) and D—*** *p* < 0.001, ** *p* < 0.01, * *p* < 0.5, significant differences between treatment with ellipticine or VPA alone and their combination (ANOVA with post-hoc Tukey HSD Test). Panels (**C**,**D**)—^∆∆∆^ *p* < 0.001, ^∆∆^ *p* < 0.01, ^∆^ *p* < 0.5, significant differences between VPA treatment compared to control (ANOVA with post-hoc Tukey HSD Test).

**Figure 2:**
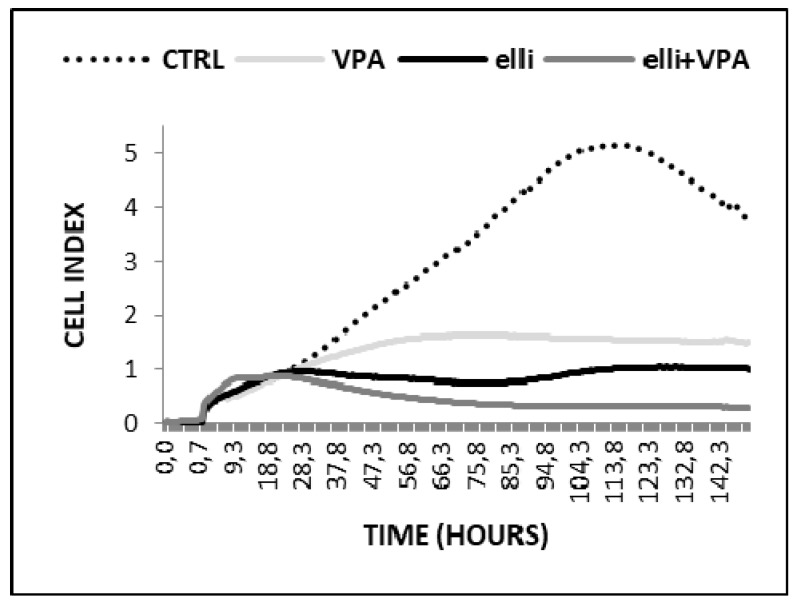
Cell index of UKF-NB-4 cells affected by 5 µM ellipticine (elli), 1 mM valproic acid (VPA) and their combination. Representative data from one of three independent experiments are shown.

Cytotoxicity of ellipticine, VPA and their combination was evaluated in UKF-NB-4 and SH-SY5Y NBL cells by the MTT method (Figure 1) and the real-time impedance-based platform xCELLigence (Figure 2). UKF-NB-4 and SH-SY5Y cells were exposed to increasing amounts of ellipticine in the presence of 1 mM VPA (Figure 1A,B) or to increasing amounts of VPA and 5 µM ellipticine (Figure 1C,D). Our results indicated that ellipticine was toxic to both UKF-NB-4 and SH-SY5Y cells, but that its toxic effect was lower in SH-SY5Y cells than in UKF-NB-4 cells; the IC_50_ values were 1.88 ± 0.13 µM and 1.27 ± 0.28 µM, respectively. In contrast, VPA was less toxic than ellipticine in NBL cells, but caused a significant decrease in cell viability at concentrations ≥0.5 and 2 mM in UKF-NB-4 and SH-SY5Y cell lines, respectively (Figure 1). When cells were treated with both drugs in combination, ellipticine cytotoxicity was higher and this effect was more pronounced in UKF-NB-4 NBL cells. This result demonstrated that VPA potentiated the cytotoxicity of ellipticine.

**Figure 3:**
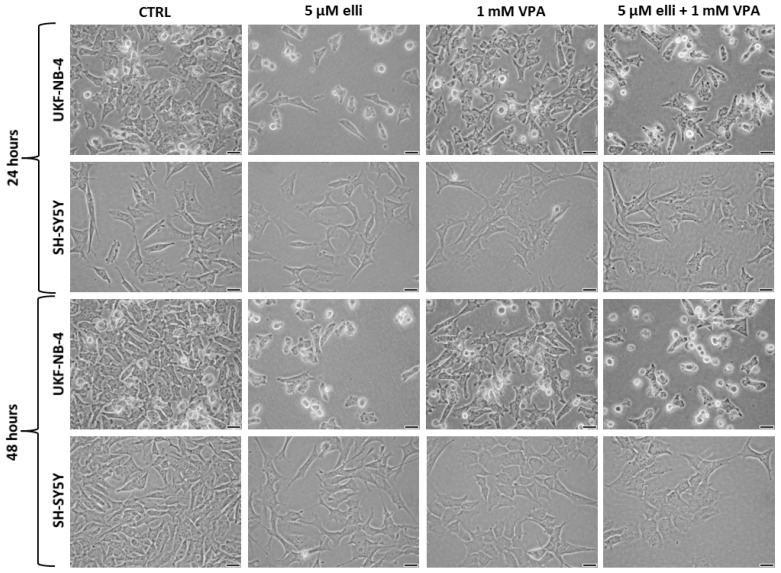
The effect of 24- and 48-h treatment of UKF-NB-4 and SH-SY5Y cells with 5 µM ellipticine, 1 mM valproic acid (VPA) and their combination on the morphology of these neuroblastoma cells (×200). As shown in this figure, VPA changes the morphology of SH-SY5Y cells. Bars at the bottom-right corners = 50 µm.

The cytotoxic potency of 5 µM ellipticine in the presence of 1 mM VPA was also increased in UKF-NB-4 cells when cell growth was analyzed using the xCELLigence system (Figure 2). The results shown in Figure 2 indicated that the UKF-NB-4 cell line cultivated with VPA grow slowly up to ~56 h of culture; their cell index did not increase after this time of cultivation. UKF-NB-4 cells exposed to ellipticine grow exponentially up to 28 h in culture, but after this time period their growth slowed down before it increased again after 75 h in culture. The highest cytotoxicity in UKF-NB-4 cells was observed by co-treatment of ellipticine with VPA; the value of cell index was lowered almost to zero (Figure 2). The xCELLigence system could not be utilized to study the effects of VPA and ellipticine in SH-SY5Y cells, because VPA led to alterations in cell morphology (Figure 3). Alterations in a cell status, i.e., cell morphology, lead to changes in values of cell index.

**Figure 4:**
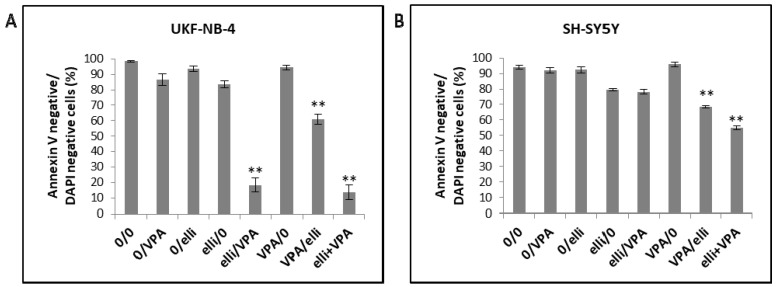
Viability of UKF-NB-4 (**A**) and SH-SY5Y (**B**) cells after incubation with 5 μM ellipticine (elli) or 1 mM valproic acid (VPA) and their various combinations. Experimental treatment conditions are described in Table 1. Values are mean ± SD from three independent experiments. ** *p* < 0.01 as compared to elli/0 group (ANOVA with post-hoc Tukey HSD Test).

**Figure 5:**
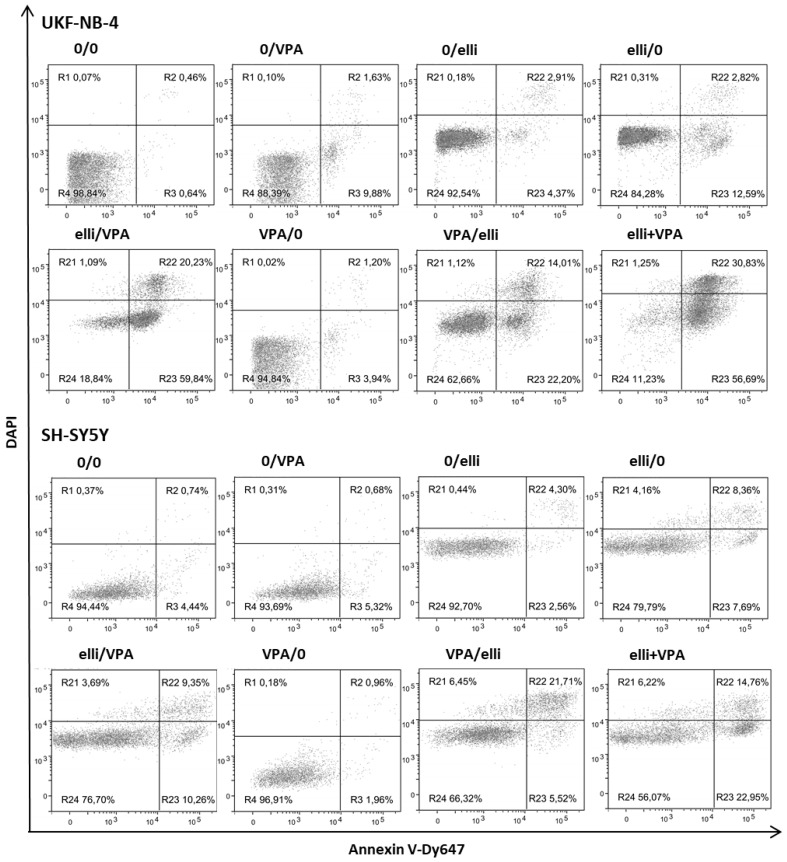
Induction of apoptosis in UKF-NB-4 and SH-SY5Y cells exposed to 1 mM valproic acid (VPA), 5 µM ellipticine (elli) and their combinations. Experimental treatment conditions are described in Table 1. Control cells (0/0) are incubated in medium without drugs. Apoptosis was measured using Annexin V-Dy647/DAPI labeling. Representative data from one of three independent experiments are shown.

Next we examined the development of apoptosis in UKF-NB-4 and SH-SY5Y cell lines after their exposure to ellipticine, VPA and the combination of both drugs. Since the influence of the combination of cytostatic drugs with HDAC inhibitors could be affected by the treatment conditions [15,20,25,26], several in vitro treatment schedules of ellipticine and VPA were utilized (Table 1). Apoptosis development in cells cultured under these different experimental conditions is demonstrated in Figure 4 and Figure 5.

Exposure of UKF-NB-4 NBL cells to 5 µM ellipticine or 1 mM VPA-induced only low levels of apoptosis (Figure 4A and Figure 5A) and cell viability was >80% after 24 or 48 h. In contrast, combined exposure of cells to ellipticine and VPA led to significant development of apoptosis (Figure 4A and Figure 5A). The sensitization of NBL cells to ellipticine by VPA depended on the sequence of drug exposure; cell viability of UKF-NB-4 cells treated first with VPA and then with ellipticine was ~60%, but the strongest potentiating effect was detected when cells were pretreated with ellipticine before VPA exposure or after co-treatment of ellipticine with VPA; cell viability was less than 20% (Figure 4A and Figure 5A).

Exposure of the SH-SY5Y cell line to 5 µM ellipticine or 1 mM VPA also-induced apoptosis (Figure 4B and Figure 5B). However, SH-SY5Y cells were less sensitive to VPA when combined with ellipticine than UKF-NB-4 cells. Cell viability of SH-SY5Y cells incubated first with ellipticine and then with VPA was similar to those cells incubated with ellipticine alone (~80%). The lower sensitivity of SH-SY5Y cells compared to UKF-NB-4 cells observed here corresponds to the results obtained in the MTT assay (see Figure 1). The strongest potentiating effect was detected after co-treatment of cells with ellipticine and VPA together (cell viability ~56%), but pre-treatment of cells with VPA also increased ellipticine toxicity (Figure 4B and Figure 5B).

Computational analysis of cell survival was calculated using the CompuSyn software (ComboSyn, Inc., Paramus, NJ, USA) [48,49] in order to evaluate whether activities of VPA and ellipticine are synergistic. The value of the effect of VPA combined with ellipticine was calculated and expressed as combination index (CI). Values for CI of less than 0.90 indicate that drugs act synergistically. CIs between 0.70 and 0.89 indicate a moderate drug synergism, whereas values of 0.9 or greater demonstrate that the tested drugs act non-synergistically [48,49]. The calculated combination index for the simultaneous influence of 5 µM ellipticine and 1 mM VPA in the UKF-NB-4 cells was less than 0.1, which demonstrates a very strong synergism of such combined treatment. In the SH-SY5Y cell line the combination treatments of ellipticine and VPA were also strongly synergistic at different combination concentrations, especially at higher concentration than 5 µM ellipticine (CI = 0.14).

**Figure 6:**
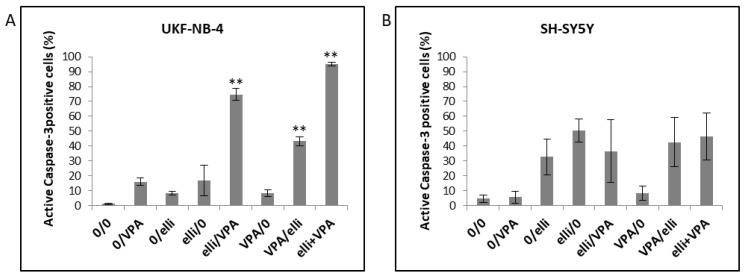
The percentage of UKF-NB-4 (**A**) and SH-SY5Y (**B**) cells with active caspase-3 after treatment with 1 mM valproic acid (VPA), 5 µM ellipticine (elli) and their combinations for 48 h. Experimental treatment conditions are shown in Table 1. Values represent mean ± SD from three independent experiments. ** *p* < 0.01 as compared to elli/0 group (ANOVA with post-hoc Tukey HSD Test).

Utilizing flow cytometry to determine the percentage of cells with active caspase-3, the caspase essential for activation of one of the primary apoptotic signaling pathways [50,51] leading to cell death [52], we showed that the above-mentioned treatment conditions are connected with apoptosis (Figure 6). Treatment of UKF-NB-4 cells simultaneously with 5 µM ellipticine and 1 mM VPA led to the highest amount of cells with active caspase-3. The second most effective treatment leading to cell death in UKF-NB-4 cells was treatment of cells with ellipticine followed by VPA treatment (Figure 6A). Such results demonstrate that apoptosis initiated by ellipticine in the UKF-NB-4 cells is caused by the activation of caspase-3 and that this effect is increased by co-treatment with VPA. In contrast, the SH-SY5Y cell line was less sensitive to VPA and ellipticine exposure than the UKF-NB-4 cell line (Figure 6B). There were essentially no differences between the amounts of SH-SY5Y cells with the active caspase-3 after pre-treatment, post-treatment or co-treatment with VPA and ellipticine.

### 2.2. VPA and Ellipticine Produce Different Effects on the Cell Cycle Distribution in UKF-NB-4 and SH-SY5Y Cells

**Figure 7:**
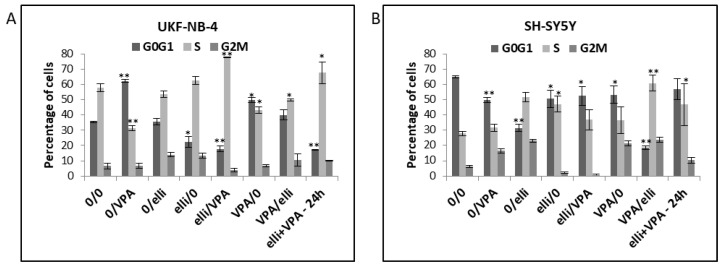
Cell cycle analysis in UKF-NB-4 (**A**) and SH-SY5Y (**B**) cells after exposure to 5 µM ellipticine (elli) and 1 mM valproic acid (VPA) and their various combinations. Experimental treatment conditions are shown in Table 1. Values represent mean ± SD from three independent experiments. ** *p* < 0.01, * *p* < 0.05 as compared to 0/0 group (ANOVA with post-hoc Tukey HSD Test).

In further experiments we studied the influence of exposure of UKF-NB-4 and SH-SY5Y cells to ellipticine and VPA on the cell cycle distribution in these cells. The reason was that combined effects of both compounds on changes in cell cycle have not been characterized as yet. Compared to controls, cells exposed to VPA resulted in increased G0/G1 arrest in the UKF-NB-4 cells but not in the SH-SY5Y cell line (Figure 7). Induction of the G0/G1 phase arrest by VPA is consistent with results found in our previous study [30]. In contrast, compared to controls, both UKF-NB-4 (Figure 7A) and SH-SY5Y cells (Figure 7B) accumulated in the S phase of cell cycle when treated with ellipticine in combination with VPA. This finding indicates that influencing of S phase of cell cycle by ellipticine with VPA is the predominant effect, which diminishes the influence of VPA on the arrest of G0/G1 phase.

### 2.3. Acetylation of Histones H3 and H4 in UKF-NB-4 and SH-SY5Y Cells Exposed to VPA, Ellipticine and Their Combination

In the next phase of our study, we studied the changes in acetylation of histones H3 and H4 in UKF-NB-4 and SH-SY5Y cell lines exposed to VPA, ellipticine and both drugs in a combination.

**Figure 8:**
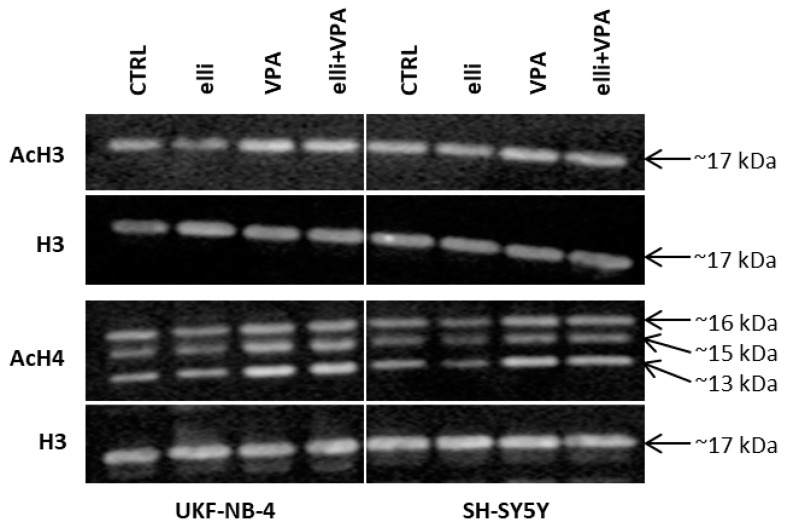
Western blot analysis of acetylated histones H3 (AcH3) and H4 (AcH4) in extracts from UKF-NB-4 and SH-SY5Y cells treated with 5 µM ellipticine (elli), 1 mM VPA and their combination. Histone H3 was used as loading control. Representative data from one of three independent experiments are shown.

As expected exposure of UKF-NB-4 and SH-SY5Y cells to 1 mM VPA resulted in an increase in acetylation of these histones in both NBL lines (Figure 8) that confirmed its HDAC inhibition effects. No influence of 5 µM ellipticine on histone acetylation was found demonstrating the absence of HDAC inhibitory efficiency of ellipticine. Treatment of both NBL cell lines with ellipticine together with VPA slightly elevated the degree of histone H3 and H4 acetylation, too, mainly in the UKF-NB-4 cell line. This phenomenon was in line with increased toxic effects of ellipticine in these cells (compare Figure 1 and Figure 4).

### 2.4. The Effect of VPA on Ellipticine-Induced DNA Damage in UKF-NB-4 and SH-SY5Y Cells

As found in previous studies, the DNA-damaging mechanisms leading to ellipticine cytotoxicity are based, beside DNA intercalation, mainly on the inhibition of topoisomerase II generating double-strand DNA breaks [38] and predominantly on the generation of covalent DNA adducts by ellipticine metabolites formed during its enzymatic activation [31,33,39,40,41,42,43,44,45]. Therefore, we examined how VPA affects the major DNA-damaging mechanism of the ellipticine action in UKF-NB-4 and SH-SY5Y cell lines.

**Figure 9:**
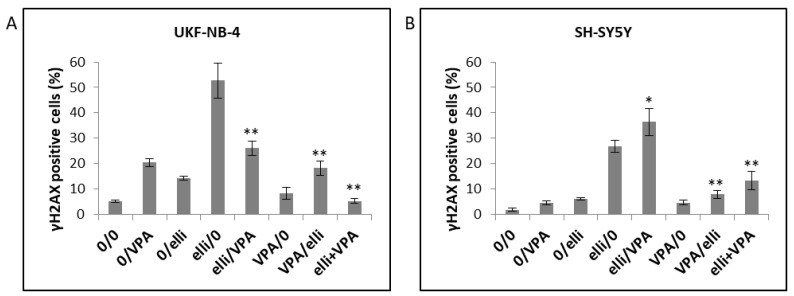
Analysis of phosphorylated H2AX in UKF-NB-4 (**A**) and SH-SY5Y (**B**) cells induced by 5 µM ellipticine (elli), 1 mM valproic acid (VPA) and their various combinations. Experimental treatment conditions are described in Table 1. Values represent mean ± SD from three independent experiments. * *p* < 0.05, ** *p* < 0.01 as compared to elli/0 group (ANOVA with post-hoc Tukey HSD Test).

Phosphorylation of histone H2A on serine 139, assigned γH2AX, by kinase enzymes sensing double-strand DNA breaks is considered to be a sensitive marker of this type of DNA damage [53,54]. NBL cells were cultured in medium containing 1 mM VPA, 5 µM ellipticine or their combinations, and the amounts of γH2AX were determined by flow cytometry (Figure 9). The percentage of cells with γH2AX did not correlate with Annexin V positive/DAPI positive cells in either NBL cell lines (compare Figure 4 and Figure 9). Hence, the effect of VPA leading to increased ellipticine-induced cytotoxicity seems to be caused mainly by mechanisms other than the induction of DNA double-strand-breaks.

**Figure 10:**
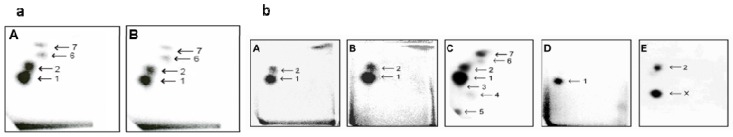
Autoradiographic profile of ^32^P-labeled ellipticine-derived DNA adducts generated in UKF-NB-4 (**aA**) and SH-SY5Y (**aB**) cells. DNA adduct pattern generated in calf thymus DNA by ellipticine after its activation with CYP3A4 in the presence of NADPH without (**bA**) and with cytochrome *b*_5_ (CYP3A4: cytochrome *b*_5_ of 1:5) (**bB**) (42); DNA of male rat liver treated with 40 mg/kg body weight ellipticine (**bC**) (31); from calf thymus DNA reacted with 13-hydroxyellipticine (**bD**) (39) or 12-hydroxyelipticine (**bE**) (40). Analyses were carried out using the nuclease P1 enrichment version of the ^32^P-postlabeling method. Adduct spots 1–7 correspond to the ellipticine-derived DNA adducts and were assigned as previously described [31].

Because ellipticine-mediated cytotoxicity is predominately linked to covalent DNA adducts formed during enzymatic activation of ellipticine catalyzed by CYP enzymes and peroxidases, we analyzed the effects of VPA on generation of ellipticine-DNA adducts using the ^32^P-postlabeling technique. As shown in Figure 10a and Table 2 up to four DNA adducts were determined by ^32^P-postlabeling in cells exposed to ellipticine alone or in combination with VPA. No DNA adducts were found in VPA-treated cells [23]. Adduct 1 that was predominantly formed in both NBL cell lines (Figure 10(aA,aB)), is generated from the ellipticine metabolite 13-hydroxyellipticine (Figure 10(bD)) which is mainly formed by CYP3A4 and this enzyme in the presence of cytochrome *b*_5_ (Figure 10(bA,bB)) [42,43,55]. Adduct 2 is generated from 12-hydroxyellipticine (Figure 10(bE)) [31,40]. Adducts 6 and 7 were detected in NBL cells only as minor adduct products (Figure 10a and Table 2). They are also generated in vivo in rats exposed to ellipticine (Figure 10(bC)) [31,41] but they have not yet been structurally identified.

**Figure 11:**
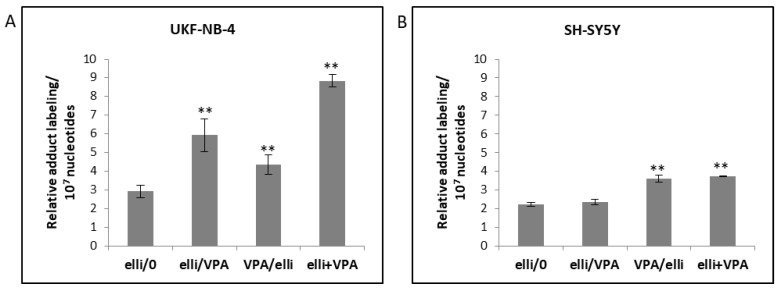
Total levels of ellipticine-derived DNA adducts formed in UKF-NB-4 (**A**) and SH-SY5Y (**B**) cells after treatment with 5 µM ellipticine (elli) and with this drug combined with 1 mM valproic acid (VPA) determined by ^32^P-postlabeling. Experimental treatment conditions are described in Table 1. Relative adduct labeling is expressed as adducts per 10^7^ normal nucleotides. Values represent mean ± SD from three independent experiments. ** *p* < 0.01 as compared to elli/0 group (ANOVA with post-hoc Tukey HSD Test).

The amounts of ellipticine-derived DNA adducts were mainly increased in UKF-NB-4 cells when pre-treated with ellipticine before VPA exposure or when cells were exposed to both drugs in combination relative to ellipticine alone. Significant higher amounts of ellipticine-derived DNA adducts were also determined in SH-SY5Y cells after co-treatment with ellipticine and VPA compared to ellipticine treatment alone (Figure 11 and Table 2). Total amounts of ellipticine-DNA adducts strongly correlated with cell viability in both UKF-NB-4 and SH-SY5Y cells (*p* < 0.01) as detected by Annexin V/DAPI staining (compare Figure 4 and Figure 11). These results confirmed previous findings that the cytotoxic action of ellipticine is based mainly on generation of covalent DNA adducts [33] and that the increase in ellipticine cytotoxicity by VPA is mainly mediated by the enhanced ellipticine-DNA adduct formation.

## 3. Discussion

Our study aimed to develop strategies for how to improve the poor response of high-risk NBL to general treatment regimens. One of the promising regimens effective against some tumor cells including NBL use DNA-damaging cytostatics such as doxorubicin, etoposide, or cisplatin in combination with several HDAC inhibitors (for a review see [15,20,22,23,24,25,26,27,28,56]). Therefore, we continued to investigate the effects of the HDAC inhibitor VPA combined with the DNA-damaging drug ellipticine in NBL cells. We employed two types of NBL cells differing in the amplification of the *N-myc* gene, namely UKF-NB-4 cells with and SH-SY5Y cells without *N-myc* amplification), because the status of this gene affects NBL biology [46]. Previously, it was found that over-expression of *N-myc* sensitizes neuroblastomas to death-receptor-induced apoptosis [57,58]. Of all genetic aberrations identified, *N-myc* amplification was the most important prognostic factor that showed a poor prognosis of NBL [1,30,59]. Therefore, our study might also help to understand how amplification of *N-myc* leads to activations of apoptotic pathways of the DNA-damaging drug ellipticine and its effectiveness.

Our results confirm previous findings that VPA utilized at the clinically relevant doses (e.g., 1 mM) can potentiate the cytotoxicity and caspase-3-mediated induction of apoptosis in NBL cells mediated by DNA-damaging chemotherapeutics such as etoposide, cisplatin [25,26] and ellipticine (present study). SH-SY5Y cells responded less sensitive to an increase in ellipticine-induced cytotoxicity mediated by VPA than UKF-NB-4 cells. Likewise, caspase-3-induced apoptosis was lower in SH-SY5Y than in UKF-NB-4 cells. Therefore, caspase-3 is required for apoptosis triggered by ellipticine and combination of ellipticine with VPA sensitized NBL cells with *N-myc* amplification. Furthermore, analyzing the values of CI, a strong synergistic effect of VPA combined with ellipticine was demonstrated in both NBL cell lines.

We investigated two types of DNA damage caused by ellipticine, namely ellipticine-mediated DNA double-strand-breaks and generation of ellipticine-derived DNA adducts and how VPA impacts on ellipticine-induced DNA damage in NBL cells. In the UKF-NB-4 cell line we did not find any proof for the increase in ellipticine-derived H2AX phosphorylation, which is considered to be a marker of DNA double-strand breaks, when ellipticine was used in combination with VPA (see Figure 9). H2AX phosphorylation increased in both NBL cells treated with ellipticine, confirming that ellipticine might participate in DNA damage by the DNA double-strand breaks mechanism. However, the degree of H2AX phosphorylation did not correlate with ellipticine cytotoxicity in the presence of VPA. Therefore, inhibition of topoisomerase-II by ellipticine which leads to DNA double-strand-break formation seems not to be the main mechanism by which VPA potentiates ellipticine toxicity in UKF-NB-4 cells. Instead we showed that increased ellipticine cytotoxicity caused by VPA correlated with elevated amounts of ellipticine-DNA adducts formed in these cells. Covalent modification of DNA by ellipticine was also found in SH-SY5Y cells and corresponded to ellipticine-induced cytotoxicity, but effects were less pronounced than in UKF-NB-4 cells. Therefore, the generation of DNA adducts by ellipticine seems to be the predominant mechanism by which ellipticine induces cytotoxicity in NBL cells, and ellipticine toxicity is further increased VPA treatment. Moreover, this process seems to depend on the amplification status of the *N-myc* oncogene in NBL cells, because both tested NBL cell lines differing in *N-myc* amplification responded differently to ellipticine and VPA treatment.

Despite our findings, the exact mechanism(s) of the synergistic effects of VPA and other HDAC inhibitors on efficiency of chemotherapeutic drugs such as ellipticine to induce DNA damage are still a matter of debate. It has been suggested that HDAC inhibitors improve elevated lysine acetylation in nucleosomal histones which are considered to relax chromatin, thereby allowing increased approach of transcription factors and DNA-damaging compounds to DNA [15,20]. In our study, enhanced toxicity of ellipticine influenced by VPA was found to depend on the acetylation status of histones H3 and H4; this HDAC inhibitor slightly enhanced the levels of acetylated histones. Moreover, the increased amounts of acetylated histones corresponded to an increase in formation of covalent ellipticine-derived DNA adducts, which finally leads to the sensitization of NBL cells (mainly the UKF-NB-4 cell line) to ellipticine.

However, it is important to underline that the sequence of ellipticine and VPA treatment is crucial to maximize the sensitivity of NBL cells to ellipticine by VPA. The HDAC inhibitor enhances the cytotoxic effects of ellipticine only when administered simultaneously, or when cells were exposed to ellipticine prior to VPA treatment. These treatment regimens were efficient to decrease the viability of NBL cells, to elevate caspase-3 activation and to enhance ellipticine-derived DNA adduct formation. In contrast, exposition of cells to VPA before exposure to ellipticine was not able to further enhance ellipticine-induced cytotoxicity or covalent DNA modification by this anticancer agent. Our results indicate that such DNA damage is essential for the promotional influence of VPA, arguing against the hypothesis that relaxed chromatin elevates the accessibility of DNA-damaging drugs to DNA [15,20]. Likewise, the finding that DNA relaxation is not essential for the synergy of the two HDAC inhibitors belinostat and romidepsin when tested with the DNA-damaging chemotherapeutics cisplatin and etoposide was also determined previously [56]. Therefore, our results indicate that alterations of the DNA structure due to covalent modification by ellipticine metabolites (i.e., the generation of ellipticine-derived DNA adducts) might elevate accessibility of at least some nucleosomal core histones to acetylation. This can consequently result in the transcription of several genes responsible for DNA repair or apoptosis. However, this hypothesis awaits further examination.

As shown in this and several previous studies [20,23,25,26,28], the most important challenge in conducting a clinical trial when combining HDAC inhibitors such as VPA with DNA-damaging drugs such as doxorubicin, etoposide, cisplatin or ellipticine will be to mediate increased DNA damage in the cancer tissue leading to tumor cell death. Our findings indicate that treatment of cells with VPA synergizes cytotoxicity of DNA-damaging agent ellipticine in NBL cells, mainly in the high-risk UKF-NB-4 cells with *N-myc* gene amplification. Further, our results shown here and those of previous studies [25,26] demonstrate that concomitant exposure to chemotherapeutic drug such as ellipticine (present study), cisplatin or etoposide [25,26] with VPA as well as the procedure where the cells were treated with chemotherapeutic prior to VPA treatment are both very effective treatment regimen which supports the development of clinical trials utilizing such combinations in neuroblastomas in children. NBL which frequently occur in children, are very rare in adults. The diagnosis in adults is difficult at an early stage as symptoms are rarely evident until the disease has metastasized. Multidisciplinary efforts for each patient should be undertaken with therapeutic regimens tailored to the adult patient. Therefore, a combinatorial treatment with ellipticine and VPA might be promising in children but might not be effective in adult patients. Combining treatment of DNA-damaging drugs with VPA might also diminish several problems occurring during NBL treatment of children in clinical practice such as the decrease in drug dose and temporary discontinuation of clinical treatment caused by drug toxicity.

## 4. Materials and Methods

### 4.1. Cell Cultures and Chemicals

The UKF-NB-4 cell line, derived from bone marrow metastases of recurrent high-risk NBL, was a present of Prof. J. Cinatl, Jr. (J. W. Goethe University, Frankfurt, Germany). The SH-SY5Y cells were from ATCC (Manassas, VA, USA). Because the SH-SY5Y cell line has been reported that can be partially contaminated (http://web.expasy.org/cellosaurus/CVCL_0019), which could influence their responses to treatment, we verified them as cells of human origin (data not shown). Valproic acid sodium salt (VPA) and ellipticine were obtained from Sigma Chemical Co. (St. Louis, MO, USA). All additional chemicals utilized in the study were of analytical purity or better. Tested NBL cell lines were cultivated at 37 °C and 5% CO_2_ in Iscove’s modified Dulbecco’s medium (IMDM) with 10% fetal bovine serum (both Life Technologies, Carlsbad, CA, USA). Cell lines were cultivated for at least 48 h with studied drugs as this time basically correlated with the time for two cycles of cell division [30]. Furthermore, this time is sufficient for the drugs investigated in the present work to enter the studied cells, influence cell cycle and cause apoptosis [22,24,25,26].

### 4.2. 3-(4,5-Dimethylthiazol-2-yl)-2,5-diphenyltetrazolium Bromide (MTT) Assay

Cytotoxicity of ellipticine and VPA in UKF-NB-4 or SH-SY5Y cells cultured in the exponential growth was determined in a 96-well plate format. To obtain dose-response curves, cells were cultivated in 100 μL of medium using a density of 10^4^ cells per well. In order to examine the effect of ellipticine, VPA and their combination on the tested NBL cell lines, cells were treated with 0.04–20 μM ellipticine and 0.16–8 mM VPA. Viability of cells was determined using the MTT test as shown previously [23,60,61]. In brief, the MTT solution (2 mg/mL in PBS) was added to cells after 48 h of their incubation at 37 °C in 5% CO_2_, the plates were incubated for 3 h and cells lysed in solution of 20% of SDS containing 50% *N*,*N*-dimethylformamide (pH 4.5). The absorbance at 570 nm was determined for each well by multiwell ELISA reader Versamax (Molecular Devices, Sunnyvale, CA, USA). The values of IC_50_ were determined using at least 3 independent measurements utilizing the linear regression of the dose-log response curves by SOFTmaxPro software.

### 4.3. Annexin V/DAPI Double Staining Assay

In order to detect apoptosis, Annexin V-Dy647 (Apronex s.r.o, Jesenice u Prahy, Czech Republic) was utilized in the procedure described in manufacturer’s instructions and cells were analyzed employing flow cytometry (LSR II, BD, Franklin Lakes, CA, USA). In brief, 8 × 10^5^ UKF-NB-4 or SH-SY5Y cells were plated in 60 mm dishes and exposed to tested chemotherapeutics, VPA (dissolved in an IMDM medium), ellipticine (dissolved in dimethyl sulfoxide [DMSO]; the final volume of DMSO did not exceed 0.5%), or these drugs in combinations. After treatment with the drugs for 48 h, cells were washed with cold PBS, trypsinized and collected by centrifugation. The tested cells were additionally re-suspended in 100 μL of Annexin binding buffer containing 1 μL of Annexin V-Dy647 and 1 μL of 4′,6-diamidino-2-phenylindole, dihydrochloride (DAPI, ThermoFisher Scientific, Waltham, MA, USA). Thereafter, cells were mildly mixed and incubated for 15 min under the ambient temperature in the dark. Binding buffer (1 mL) was added to every tube and fudged. Pellets of collected cells were re-suspended in the same buffer, measured using a LSR II flow cytometer (BD, Franklin Lakes, CA, USA) and tested using FlowLogic software (Inivai Technologies, Mentone, Australia).

### 4.4. Real-Time Monitoring of Cell Viability

The xCELLigence RTCA DP Instrument (ACEA Bioscience Inc., San Diego, CA, USA) placed in a humidified incubator at 37 °C and 5% CO_2_ was utilized for real-time label free monitoring of cell viability [62]. UKF-NB-4 and/or SH-SY5Y cell lines (15,000 cells) were seeded into wells of 16-well plates for impedance-based detection. Every treatment situation (control, 1 mM VPA, 5 µM ellipticine and the mixture of 1 mM VPA with 5 µM ellipticine) was analyzed in duplicate. Cell index (CI) was measured every 30 min for 140 h and results were monitored using the supplied RTCA software.

### 4.5. Detection of Active Caspase-3

In order to recognize cells possessing active caspase-3, 8 × 10^5^ UKF-NB-4 and/or SH-SY5Y neuroblastoma cell lines were plated in 60 mm dishes and exposed to tested chemotherapeutics or their combinations for 48 h. The amounts of active caspase-3 positive cells were determined employing the cleaved caspase-3 (Asp175) (D3E9) rabbit mAb (Alexa Fluor^®^ 647 Conjugate) #9602 (Cell Signaling Technology, Danvers, MA, USA). Briefly, after treatment, cells were washed with cold PBS, trypsinized and collected by centrifugation. Pellets of cells were washed with PBS and after spinning fixed in 4% paraformaldehyde for 10 min. Cell pellets were then again washed with PBS and permeabilized by adding 90% methanol for 1 h at ‒20 °C. Pellets were subsequently washed 3 times with 1 mL 0.5% bovine serum albumin (BSA) in PBS, cells re-suspended in 100 µL of diluted antibody and incubated for 1 h at laboratory temperature. Re-suspended washed cells were measured using a LSR II flow cytometer (BD, Franklin Lakes, CA, USA) and tested using FlowLogic software.

### 4.6. Cell Cycle Analysis

In order to evaluate cell cycle distribution, 8 × 10^5^ cells were plated in 60 mm dishes and exposed to individual drugs or their combinations for various incubation periods. After exposure, the cells were collected by trypsinization, washed by PBS and fixed with 4% paraformaldehyde for 10 min. Thereafter these cells were permeabilized with 90% methanol and incubated in ‒20 °C a minimum of 1 h. Samples were additionally incubated in DAPI (1 μg/mL) solution in dark under the laboratory temperature for 30 min, washed with PBS, and measured by flow cytometry employing LSR II flow cytometer and analyzed with FlowLogic software.

### 4.7. Determination of Contents of Acetylated Histones H3 and H4

To determine the histone H3 and H4 acetylation status, 5.4 × 10^6^ cells were plated in 100 mm dishes, cultivated and then exposed to 5 μM ellipticine, 1 mM VPA and with both compounds in combination for 48 h. Cells were harvested and histones isolated using acid extraction followed by precipitation of histones using trichloracetic acid (TCA) [63]. Amounts of proteins were measured as described previously [64], employing the DC Protein Assay (Bio-Rad, Hercules, CA, USA). Histones (5 µg) were separated by electrophoresis on 16% polyacrylamide gels, transferred onto nitrocellulose membrane and incubated with 5% non-fat milk to block non-specific binding. The nitrocellulose membranes were subsequently treated with specific rabbit polyclonal anti-acetyl-histone H3 (1:2000; Upstate Biotechnology Inc., Lake Placid, NY, USA) and anti-acetyl-histone H4 (1:2000; Merck Millipore, Billerica, MA, USA) antibodies overnight at 4 °C. Thereafter they were washed and treated with europium-labeled goat anti-rabbit and goat anti-mouse secondary antibodies (1:5000; Molecular Devices, Sunnyvale, CA, USA) and the antigen-antibody complex was visualized by SpectraMax i3x Multi-Mode Detection Platform according to the manufacturer’s instructions (Molecular Devices, Sunnyvale, CA, USA). The anti-histone H3 antibody (1:8000; Merck Millipore, Billerica, MA, USA) was employed as a loading control.

### 4.8. Determination of Histone H2AX Phosphorylation Status

To evaluate phosphorylation of histone H2AX, 8 × 10^5^ neuroblastoma cells were plated in 60 mm dishes and exposed to tested chemotherapeutics or their combinations. After treatment cells were washed and subsequently fixed in 4% formaldehyde in PBS for 10 min. Thereafter they were washed by PBS, re-suspended in ice cold 90% methanol and incubated for 1 h at −20 °C. After washing three times with wash buffer (PBS containing 0.5% BSA and 0.2% Triton X) cells were incubated in 50 μL of wash buffer containing 5 µL of pH2AX antibody (Alexa Fluor^®^ 647 anti-H2AX-Phosphorylated (Ser139), Biolegend, San Diego, CA, USA) for 60 min at 4 °C. Then cells were washed, measured using a LSR II (BD, Franklin Lakes, CA, USA) and analyzed with FlowLogic software.

### 4.9. Detection of Ellipticine-DNA Adducts by ^32^P-Postlabeling

5.4 × 10^6^ cells were plated in 100 mm dishes and exposed to 5 µM ellipticine or 5 µM ellipticine with 1 mM VPA for 48 h. Cells were harvested after trypsinizing and washed twice with 5 mL of PBS yielding a cell pellet that was stored at ‒80 °C until DNA isolation. DNA was isolated by a standard phenol-chloroform extraction method as described [23,31,40,65,66]. Ellipticine-DNA adduct were detected and quantified utilizing the nuclease P1 enrichment version of the ^32^P-postlabeling assay as described previously for in vitro [23,31,40,65,66] and in vivo [31,41] analyses.

### 4.10. Statistical Analysis

Data are shown as averages ± SD. ANOVA with post-hoc Tukey HSD Test was utilized when comparing the situations. *p* < 0.05 was considered as significant. Significances of the statistical analyses are shown in individual Figures and described in their legends.

## 5. Conclusions

In conclusion, VPA sensitizes NBL cells to ellipticine toxicity. This sensitization was found to depend on the sequence of drug treatment; synergistic effects were only seen either after simultaneous exposure of cells to ellipticine together with VPA or when the cells were exposed to ellipticine prior to treatment with VPA. The synergistic effects of VPA with ellipticine were connected with increased H3 and H4 histone acetylation, mainly in UKF-NB-4 cells. The G0/G1 phase arrest was detected in UKF-NB-4 cells after VPA treatment, which is consistent with such an effect in various cancer cells [12,15], but not found in SH-SY5Y cells. Treatment of cells with ellipticine and VPA led to accumulation of both NBL cell lines in the S-phase of cell cycle resulting from increased DNA damage in these cells, and this effect diminishes the influence of VPA on the arrest in G0/G1 phase. Enhanced ellipticine-derived DNA adduct formation stimulated by VPA treatment was predominantly responsible for increased ellipticine cytotoxicity by VPA, indicating that the elevated accessibility of DNA to this anticancer agent is caused by VPA.

## Figures and Tables

**Table 1:** Treatment schedules of ellipticine (elli) and valproic acid (VPA) used in UKF-NB-4 and SH-SY5Y neuroblastoma cell lines.

Designation	0–24 h	24–48 h
0/0	medium	
0/VPA	medium	1 mM VPA
0/elli	medium	5 µM elli
elli/0	5 µM elli	
elli/VPA	5 µM elli	1 mM VPA
VPA/0	1 mM VPA	
VPA/elli	1 mM VPA	
elli + VPA	5 µM elli + 1 mM VPA	

elli, ellipticine; VPA, valproic acid.

**Table 2:** DNA adduct formation by ellipticine in neuroblastoma UKF-NB-4 and SH-SY5Y cell lines treated with ellipticine and this drug combined with VPA. Experimental treatment conditions are described in Table 1.

Cells	RAL (Mean ± SD/10^7^ Nucleotides) ^a^				
	Adduct 1 ^b^	Adduct 2	Adduct 6	Adduct 7	Total
**UKF-NB-4**					
elli/0	1.86 ± 0.31	0.53 ± 0.01	0.22 ± 0.04	0.30 ±0.005	2.91 ± 0.35
elli/VPA	4.42 ± 0.06 **	0.30 ± 0.02 *	0.47 ± 0.07 **	0.74 ± 0.09 *	5.93 ± 0.87 **
VPA/elli	2.72 ± 0.02 **	0.23 ± 0.06 **	0.60 ± 0.04 **	0.80 ± 0.10 **	4.35 ± 0.52 **
elli + VPA	6.46 ± 0.05 **	0.24 ± 0.02 **	1.04 ± 0.02 **	1.10 ± 0.07 **	8.84 ± 0.34 **
**SH-SY5Y**					
elli/0	1.50 ± 0.13	0.12 ± 0.01	0.26 ± 0.03	0.34 ± 0.07	2.22 ± 0.11
elli/VPA	1.43 ± 0.19	0.17 ± 0.02 **	0.37 ± 0.02 *	0.37 ± 0.06	2.34 ± 0.16
VPA/elli	2.37 ± 0.01 **	0.26 ± 0.001 **	0.37 ± 0.06 *	0.59 ± 0.07 **	3.59 ± 0.19 **
elli + VPA	2.37 ± 0.01 **	0.27 ± 0.01 **	0.45 ± 0.01 **	0.65 ± 0.006 **	3.74 ± 0.02 **

elli—5 µM ellipticine, VPA—1 mM VPA. ^a^: Relative adduct labeling. ^b^: For adduct numbers, see Figure 10. ** *p* < 0.01, * *p* < 0.05 (ANOVA with post-hoc Tukey HSD Test), significantly different from cells treated with ellipticine alone.
